# Dose-volume relationship for laryngeal substructures and aspiration in patients with locally advanced head-and-neck cancer

**DOI:** 10.1186/s13014-019-1247-7

**Published:** 2019-03-18

**Authors:** Katarina G. Petras, Alfred W. Rademaker, Tamer Refaat, Mehee Choi, Tarita O. Thomas, Barbara R. Pauloski, Bharat B. Mittal

**Affiliations:** 1 Department of Radiation Oncology, Northwestern University, Chicago, IL USA; 2 Biostatistics Department, Northwestern University, Chicago, IL USA; 3 Department of Clinical Oncology, Faculty of Medicine, Alexandria University, Alexandria, Egypt; 4 Department of Radiation Oncology, Loyola University Medical Center, Maguire Center – Room 2944, 2160 S. 1st Avenue, Maywood, IL 60153 USA; 5 Rush Copley Medical Center, 2000 Ogden Avenue, Aurora, IL 60504 USA; 6 Department of Communication Sciences and Disorders, College of Health Sciences, University of Wisconsin-Milwaukee, Enderis Hall, Room 845, 2400 E. Hartford, Avenue, Milwaukee, WI 53211 USA; 7 Department of Radiation Oncology, NMH, 251 E. Huron Street, LC-178, Chicago, IL 60611 USA; 8 Department of Biostatistics & Preventative Medicine, 680 N. Lakeshore Drive, Suite 1400, Chicago, IL 60611 USA

**Keywords:** Radiotherapy, Head-and-neck cancer, Aspiration, Larynx

## Abstract

**Background:**

Literature has shown a significant relationship between radiation dose to the larynx and swallowing disorders. We prospectively studied the dose-volume relationship for larynx substructures and aspiration.

**Methods:**

Forty nine patients with stage III/IV head-and-neck (H&N) squamous cell carcinoma were prospectively enrolled in this IRB-approved, federally funded study. All patients received IMRT-based chemoradiation therapy (CRT) and were scheduled for videofluorography (VFG) prior to CRT and at 3, 6, 9, 12, and 24 months post-CRT. Twelve laryngeal substructures were contoured in each patient: thyroid cartilage, cricoid cartilage, total epiglottis, suprahyoid epiglottis, infrahyoid epiglottis, total larynx, supraglottic larynx, subglottic larynx, glottic larynx, arytenoids, aryepiglottic (AE) folds, and glossoepiglottic fold. After exclusions, 29 patients were included in the final analysis. Incidence of aspiration at 1 year following CRT was correlated with dose-volume data to laryngeal substructures using logistic regression.

**Results:**

The median age was 54 years with 79% being non-smokers. Tumor sites included oropharynx (22), unknown primary (6), and hypopharynx (1). One year following CRT, 10/29 (34%) showed aspiration on VFG. Dose to the AE folds showed the highest correlation with aspiration at 12 months and was significant on multivariate analysis (*p* = 0.025). A mean dose cutpoint of 6500 cGy or higher to the AE folds was associated with an increased risk of aspiration at 1 year [positive likelihood ratio (+LR) 2.81, positive predictive value (PPV) 60%, negative predictive value (NPV) 92.9%, relative risk (RR) 8.4].

**Conclusions:**

In this analysis, mean dose to the AE folds was associated with an increased risk of aspiration at 1 year. However, these are hypothesis-generating data that require further research and validation in a larger patient subset.

## Background

Swallowing is a complex series of events that involves the seamless coordination of multiple muscles and nerves to bring food from the oral cavity to the stomach. Swallowing dysfunction is a known long-term toxicity of post-operative radiotherapy (RT), definitive RT, and chemoradiation therapy (CRT) used in the treatment of H&N cancer. Swallowing dysfunction has a major impact on the quality of life experienced by H&N cancer survivors and requires close examination [1, 2].

Treatment-related swallowing dysfunction results from damage to structures involved in different phases of the swallowing cycle. These include the salivary glands, tongue and oropharyngeal muscles, laryngeal-hyoid range of motion, and the upper esophagus [3]. Proper laryngeal anatomy and movement is particularly crucial to normal swallowing physiology and prevention of aspiration. Toxicity is related to RT factors such as total dose, fraction size, radiated volume, inter-fraction time interval, treatment technique, and tissue-dose compensation [4–7]. It is also impacted by the addition of chemotherapy, tumor site and stage, patient age, as well as tobacco use [8–11].

A dose-volume relationship has been shown to exist between various H&N organs and risk of swallowing dysfunction. These include the larynx, pharyngeal constrictors, and upper esophagus. Various studies have shown that dose reduction to these organs-at-risk (OARs) can help decrease the risk of long-term swallowing dysfunction [12–21]. In recent years, several contouring guidelines have been released in order to facilitate standard and accurate delineation of laryngeal OARs [22, 23].

Overall, more information is needed to determine which laryngeal substructures, when irradiated, are most associated with swallowing dysfunction. Given this, we designed a prospective study to examine the dose-volume relationship between 12 larynx substructures and long-term aspiration risk. This assessment required accurate knowledge of each patient’s baseline swallowing function and then consistent, interval post-RT follow-up to monitor clinical progress or decline over time.

## Methods

### Eligibility & Data Collection

Forty-nine patients with stage III/IV loco-regionally advanced H&N squamous cell cancer were prospectively enrolled in this IRB-approved, federally funded study from 2006 to 2011. All patients received intensity modulated radiotherapy (IMRT)-based CRT.

Patients were scheduled for videofluorography (VFG) prior to CRT and at 3, 6, 9, 12, and 24 months post-CRT. During each study the patient received an oral barium bolus, and a total of 22 temporal components of swallowing function [24] were evaluated. The VFG studies were conducted in the lateral plane according to the procedure outlined by Logemann [25] and recorded at 30 frames per second. Each swallow study was assessed for presence or absence of aspiration before, during, and after the pharyngeal swallow. Aspiration was defined as being present if any portion of the barium bolus entered the airway below the level of the true vocal folds. Aspiration may have occurred at multiple times for a swallow. Each individual patient was then classified as having aspirated if they were assessed as having aspiration present at any point (before, during, or after the pharyngeal swallow) on at least one of the multiple swallows in the VFG evaluation.

Two research technicians shared the analysis of the swallow studies for this study. Research technicians were initially trained in their accuracy until their interobserver and intraobserver reliability was at least 0.85. Once the study commenced, 10% of the analyzed swallows were chosen at random for reanalysis by both research technicians to determine inter-judge and intra-judge reliability. Lin’s Concordance Coefficient was used to quantify reliability. Average inter- and intra-observer reliability with Lin’s Concordance Coefficient were .989 and .992, respectively. Since a number of these measures play an important role in aspiration, we correlated DVH data to aspiration only.

The following 12 larynx substructures were retroactively contoured in each patient per guidelines outlined by Choi et al. [22]: thyroid cartilage, cricoid cartilage, total epiglottis, suprahyoid epiglottis, infrahyoid epiglottis, total larynx, supraglottic larynx, subglottic larynx, glottic larynx, arytenoids, aryepiglottic (AE) folds, and glossoepiglottic fold. Of these 49 patients, 13 were excluded due to not having a VFG evaluation at 12 months of follow-up (10 oropharyngeal, 3 unknown primary), 4 were excluded due to prevalent aspiration at baseline (3 oropharyngeal, 1 hypopharyngeal), and 3 were excluded due to having primary laryngeal cancer. In total, 29 patients were included in our analysis.

### Treatment technique

The details of our sequential treatment technique have been previously reported [26]. Briefly, planning target volumes (PTVs) were generated from clinical target volumes (CTVs) corresponding to areas of high, intermediate, and low-risk disease. These areas received 66–75 Gy, 56–60 Gy, and 50–56 Gy, respectively. Treatment was delivered without the use of image-guided radiotherapy (IGRT). In this paper our reported mean larynx doses are towards the higher side because during the initial years of this study, we did not utilize very strict mean dose parameters. Additionally, our larynx contours are more generous and comprehensive than what is traditionally defined as laryngeal tissue. In more recent years we restricted the mean larynx dose to < 45 Gy.

### Statistical methods

Patient demographics, tumor characteristics, and treatment modalities were compared between aspiration groups using Fisher’s exact test, except for age and total dose where the independent sample t-test was used. Mean dose to each of the 12 structures was compared between aspiration groups using logistic regression, which resulted in a receiver operating characteristic (ROC) curve. The area under the ROC curve (AUC) indicated the strength of the relationship between dose and aspiration. Multivariate analysis was performed using logistic regression.

For each substructure, a dose cutpoint was determined which maximized the sum of sensitivity and specificity. Sensitivity was defined as the percentage of aspirators whose dose to that substructure was above the cutpoint. Specificity was defined as the percentage of non-aspirators whose dose to that substructure was below the cutpoint. The positive likelihood ratio (+LR), which is sensitivity/(100-specificity), is the percentage of aspirators above the cutpoint divided by the percentage of non-aspirators above the cutpoint. Positive predictive value (PPV) was the percentage of patients above the cutpoint who aspirated. Negative predictive value (NPV) was the percentage of patients below the cutpoint who did not aspirate. Relative risk (RR) was the percentage of patients above the cutpoint who aspirated, divided by the percentage of patients below the cutpoint who aspirated. Pearson correlation coefficients were calculated between doses to pairs of structures. Inter-correlation of doses was illustrated using least squares regression and a 95% prediction interval.

## Results

Patient demographics, tumor characteristics, and treatment modalities are outlined in Table 1. There was no significant difference on these attributes between aspirators and non-aspirators. Among the final 29 patients analyzed, the median age was 54 (range 30–73). The majority of patients were male (79.3%) and non-smokers (79.3%). Tumor sites included oropharynx (22 patients), unknown primary (6 patients), and hypopharynx (1 patient). Most patients were stage IVA at the time of diagnosis (26), followed by stage III (2), and stage IVB (1). p16 status was available for 15 patients, and 14 of these were p16 positive on pathologic review. None of the patients included in this study had tumor directly infiltrating the larynx. The rate of aspiration at 1 year was 34% (10 aspirators vs. 19 non-aspirators).

**Table 1:** Patient demographics, tumor characteristics and treatment modalities

	All Patients (*n* = 29)	Aspirators (*n* = 10)	Non-aspirators (*n* = 19)	*p*-value
Median age	54 (30–73)	54 (31–67)	54 (30–73)	0.75
Dose (cGy)	7000 (6400–7500)	7175 (7000–7500)	7000 (6400–7500)	0.11
	*N* (%)	*N* (%)	*N* (%)	
Sex				
Male	23 (79.3)	7 (70.0)	16 (84.2)	0.63
Female	6 (20.7)	3 (30.0)	3 (15.8)	
Smoking				
No	23 (79.3)	6 (60.0)	17 (89.5)	0.14
Yes	6 (20.7)	4 (40.0)	2 (10.5)	
Tumor Site				
Oropharynx	22 (75.9)	9 (90.0)	13 (68.4)	0.065
Unknown Primary	6 (20.7)	0	6 (31.6)	
Hypopharynx	1 (3.5)	1 (10.0)	0	
T stage				
T0	5 (17.2)	0	5 (26.3)	0.18
T1	8 (27.6)	3 (30.0)	5 (26.3)	
T2	11 (37.9)	4 (40.0)	7 (36.8)	
T3	4 (13.8)	3 (30.0)	1 (5.3)	
T4	1 (3.5)	0	1 (5.3)	
N stage				
N1	1 (3.5)	0	1 (5.3)	0.17
N2a	3 (10.3)	0	3 (15.8)	
N2b	21 (72.4)	7 (70.0)	14 (73.7)	
N2c	4 (13.8)	3 (30.0)	1 (5.3)	
Stage				
III	2 (6.9)	1 (10.0)	1 (5.3)	0.42
IVA	26 (89.6)	8 (80.0)	18 (94.7)	
IVB	1 (3.5)	1 (10.0)	0	
Grade				
1	1 (3.5)	0	1 (5.3)	0.44
2	9 (31.0)	5 (50.0)	4 (21.1)	
3	9 (31.0)	2 (20.0)	7 (36.8)	
Unknown	10 (34.5)	3 (30.0)	7 (36.8)	
p16 status				
Positive	14 (48.3)	3 (30.0)	11 (57.9)	0.99
Negative	1 (3.4)	0	1 (5.3)	
Unknown	14 (48.3)	7 (70.0)	7 (36.8)	

**Fig. 1:**
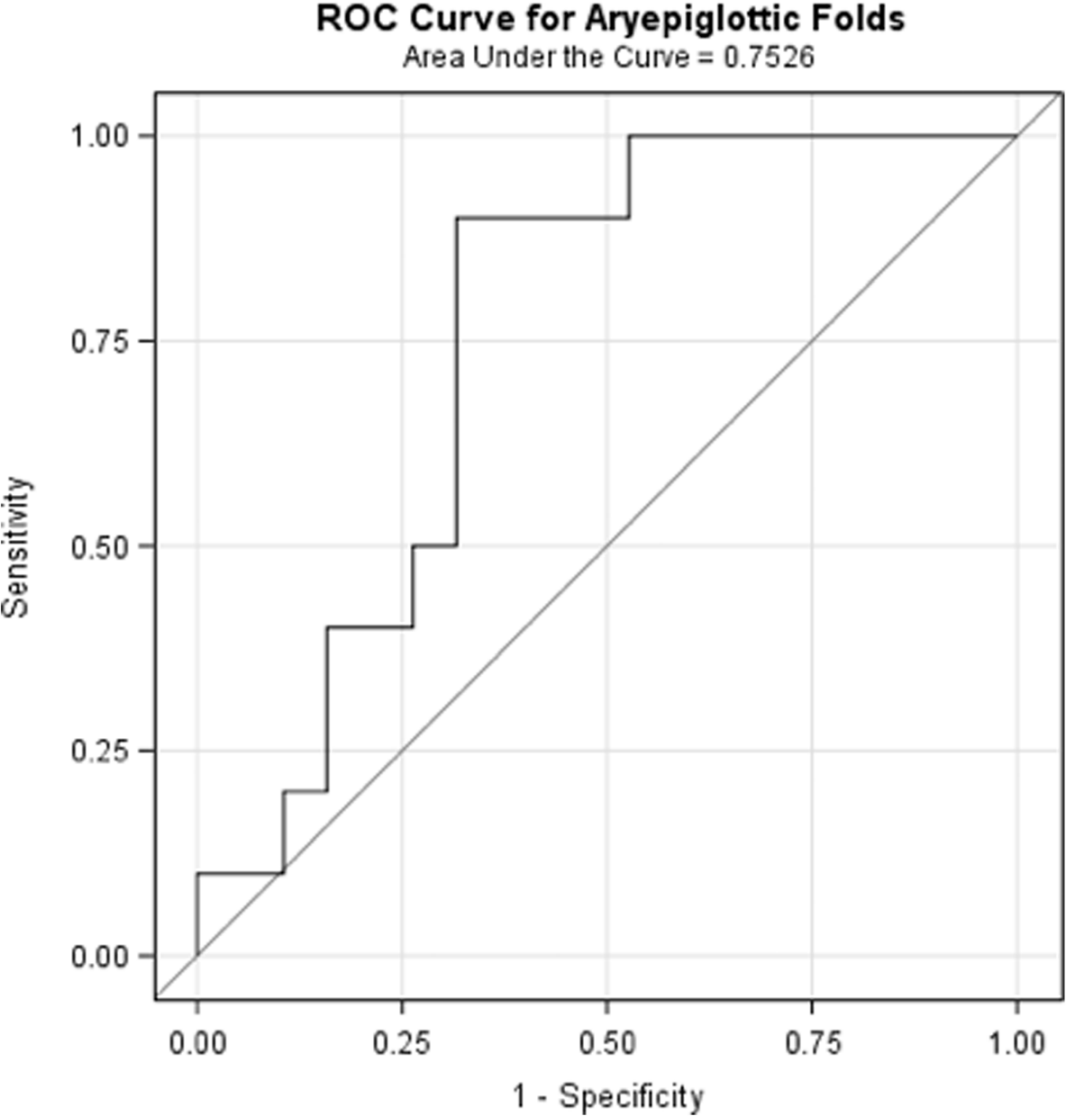
Receiver Operating Characteristic (ROC) curve relating mean dose to aryepiglottic folds with aspiration at 12 months

Table 2 outlines the results of our univariate and multivariate analysis. For each patient, the mean dose for each of the 12 larynx substructures was determined. We calculated the mean and standard deviation of these mean doses among aspirators and non-aspirators. After generating ROC curves using logistic regression, the AUC was used to determine the strength of the relationship between substructure dose and aspiration at 12 months. The AUC was highest for AE folds (Fig. 1). Univariate analysis identified multiple laryngeal substructures with significant association between RT dose and aspiration, however on multivariate analysis, only dose to the AE folds was significant (*p* = 0.025).

**Table 2:** Mean dose (cGy) by aspiration status for each laryngeal substructure

Sample size	All Patients	Aspirators	Non-aspirators	AUC	Univariate *p*-value	Multivariate *p*-value
	29	10	19			
	Mean (sd)	Mean (sd)	Mean (sd)			
Structure						
AE Folds	6606 (679)	7025 (398)	6386 (700)	0.753	0.025	0.025
Arytenoids	5832 (1070)	6419 (568)	5523 (1152)	0.737	0.045	NS
Glossoepiglottic fold	7304 (459)	7507 (289)	7197 (500)	0.668	0.096	NS
Cricoid cartilage	5722 (975)	6207 (616)	5466 (1044)	0.695	0.063	NS
Thyroid cartilage	6406 (739)	6841 (532)	6178 (741)	0.747	0.029	NS
Epiglottis	6947 (611)	7300 (406)	6761 (626)	0.747	0.032	NS
Suprahyoid epiglottis	7178 (482)	7422 (360)	7050 (497)	0.726	0.058	NS
Infrahyoid epiglottis	6487 (840)	6867 (573)	6287 (901)	0.711	0.085	NS
Total larynx	6217 (823)	6675 (504)	5977 (866)	0.747	0.038	NS
Supraglottic larynx	6573 (754)	6996 (446)	6350 (795)	0.732	0.037	NS
Subglottic larynx	5571 (1062)	6018 (638)	5336 (1175)	0.679	0.110	NS
Glottic larynx	5834 (1074)	6358 (634)	5559 (1167)	0.711	0.067	NS

*sd* Standard deviation, *AUC* Area under the receiver operating characteristic curve, *NS* Not significant

Table 3 highlights the mean dose cutpoint for each larynx substructure that maximized the sum of sensitivity and specificity for aspiration at 1 year. For example, a mean dose of 6500 cGy or higher to the AE folds was associated with an increased risk of developing aspiration at one year (+LR 2.81, PPV 60%, NPV 92.9%, RR 8.4).

**Table 3:** Dose cutpoint, sensitivity, specificity and positive likelihood ratio, positive predictive value (PPV), negative predictive value (NPV) and relative risk (RR)

	Mean dose cutpoint (cGy)	Number (%) of aspirators above cutpoint (sensitivity)	Number (%) of non-aspirators below cutpoint (specificity)	Positive likelihood ratio: sensitivity/(100-specificity)	PPV(%)^a^	NPV(%)^b^	RR^c^
Structure							
AE Folds	6500	9/10 (90.0)	13/19 (68.4)	2.81	60.0	92.9	8.4
Arytenoids	6000	8/10 (80.0)	14/19 (73.7)	3.04	61.5	87.5	4.9
Glossoepiglottic fold	7000	10/10 (100.0)	8/19 (42.1)	1.73	47.6	100.0	undefined
Cricoid cartilage	6000	7/10 (70.0)	14/19 (73.7)	2.66	58.3	82.4	3.3
Thyroid cartilage	6450	9/10 (90.0)	14/19 (73.7)	3.42	64.3	93.3	9.6
Epiglottis	7225	7/10 (70.0)	14/19 (73.7)	2.66	58.3	82.3	3.3
Suprahyoid epiglottis	6810	10/10 (100.0)	9/19 (47.4)	1.90	50.0	100.0	undefined
Infrahyoid epiglottis	6300	9/10 (90.0)	12/19 (63.2)	2.45	56.3	92.3	7.3
Total larynx	6100	9/10 (90.0)	13/19 (68.4)	2.85	60.0	92.9	8.5
Supraglottic larynx	6650	9/10 (90.0)	13/19 (68.4)	2.85	60.0	92.9	8.5
Subglottic larynx	5750	7/10 (70.0)	13/19 (68.4)	2.66	53.9	81.3	2.9
Glottic larynx	6100	8/10 (80.0)	14/19 (73.7)	2.81	61.5	87.5	4.9

^a^PPV should be greater than the a priori prevalence of aspiration which is 34.5%

^b^NPV should be greater than the a priori prevalence of no aspiration which is 65.5%

^c^RR is undefined if denominator is zero

## Discussion

This manuscript reports the results of a prospective study examining the relationship between laryngeal substructure dose and aspiration risk at 1 year among 29 patients treated definitively with CRT for locally advanced H&N cancer. All patients underwent baseline and post-treatment swallowing evaluation with VFG for at least 1-year. The rate of aspiration at 1 year was 34% (10/29 patients). We found that dose to the AE folds was most strongly correlated with 1-year aspiration risk, and this held true on multivariate analysis. Furthermore, a mean dose cutpoint of 6500 cGy to this structure was associated with an increased risk of developing aspiration at this time point. This is logical from a functional standpoint as the AE folds provide central airway protection by adducting during swallowing to prevent aspiration.

While treatment intensification for H&N cancer has improved cancer-specific outcomes, it has also increased the incidence of dysphagia and aspiration complications [27–29]. However, highly conformal dose distributions, attainable through the implementation of IMRT and on board cone beam computed tomography imaging, can be used to spare dose to critical OARs and reduce adverse toxicity [14, 30–33]. There is hope that normal tissue sparing could be further improved with the implementation of proton-based radiotherapy in appropriately selected patients [34]. Swallowing function can also be preserved and improved with timely identification of patients at risk of aspiration/dysphagia and initiation of prophylactic swallowing exercises [35, 36].

Dose to the AE folds and its impact on patient swallowing/nutrition has been identified before. A study by Dornfeld, et al. examined the outcomes of 27 patients treated with definitive IMRT for H&N cancer who were disease free for at least 1 year post-treatment [17]. The authors found that higher doses to the AE folds, false vocal cords, and lateral pharyngeal walls were correlated with a more restricted patient diet. Higher dose to the AE folds was also correlated with greater weight loss at 1 year.

When interpreting the results of this paper, several limitations should be noted. One is our relatively small sample size, especially after some patients were excluded for presence of baseline aspiration and insufficient follow-up. Additionally, no quality of life data was collected. Finally, there was heterogeneity in primary target PTV coverage and larynx doses.

The strength of this paper lies in the prospective and objective collection of its data points. Our identification of AE folds as a laryngeal substructure of interest could help streamline the radiation planning and evaluation process because, logistically, it is not practical to contour 12 laryngeal substructures for every patient. Our findings of association between mean dose to the larynx and aspiration is similar to published results by other authors [12, 13, 17]. However, our approach of using standardized methods to contour and define the larynx makes our dose-volume relationship findings unique.

## Conclusions

Overall, these are hypothesis-generating data that require further research and validation in a larger number of patients to assess the importance of dose to laryngeal substructures and future aspiration risk. Implementation of modern radiotherapy techniques, such as either IMRT or proton therapy, could be used to further reduce mean radiation doses to the larynx and critical OARs within the larynx, such as the AE folds and reduce the risk for aspiration.

## Abbreviations

AE: Aryepiglottic
AUC: Area under the curve
CRT: Chemoradiation therapy
CTV: Clinical target volume
H&N: Head and neck
IGRT: Image-guided radiotherapy
IMRT: Intensity-modulated radiotherapy
LR: Likelihood ratio
OARs: Organs-at-risk
PTV: Planning target volume
ROC: Receiver operating characteristic
RT: Radiotherapy
VFG: Videofluorography
